# Gelation or molecular recognition; is the *bis*-(α,β-dihydroxy ester)s motif an omnigelator?

**DOI:** 10.3762/bjoc.6.123

**Published:** 2010-11-18

**Authors:** Peter C Griffiths, David W Knight, Ian R Morgan, Amy Ford, James Brown, Ben Davies, Richard K Heenan, Stephen M King, Robert M Dalgliesh, John Tomkinson, Stuart Prescott, Ralf Schweins, Alison Paul

**Affiliations:** 1 School of Chemistry, Cardiff University, Main Building, Park Place, Cardiff CF10 3AT, U.K.; 2 Rutherford Appleton Laboratory, Science and Technology Research Council, Didcot, Oxfordshire OX11 0QX U.K.; 3 School of Chemistry, Bristol University, Cantock’s Close, Bristol BS8 1TS U.K.; 4 Institut Laue Langevin, 6 rue Jules Horowitz, 38042 Grenoble, Cedex 9, France

**Keywords:** gelation, inelastic neutron spectroscopy, low molar mass organogelator, neutron scattering, neutron spin-echo scattering, self-assembly

## Abstract

Understanding the gelation of liquids by low molecular weight solutes at low concentrations gives an insight into many molecular recognition phenomena and also offers a simple route to modifying the physical properties of the liquid. *Bis*-(α,β-dihydroxy ester)s are shown here to gel thermoreversibly a wide range of solvents, raising interesting questions as to the mechanism of gelation. At gelator concentrations of 5–50 mg ml^−1^, gels were successfully formed in acetone, ethanol/water mixtures, toluene, cyclohexane and chloroform (the latter, albeit at a higher gelator concentration). A range of neutron techniques – in particular small-angle neutron scattering (SANS) – have been employed to probe the structure of a selection of these gels. The universality of gelation in a range of solvent types suggests the gelation mechanism is a feature of the *bis*-(α,β-dihydroxy ester) motif, with SANS demonstrating the presence of regular structures in the 30–40 Å range. A correlation between the apparent rodlike character of the structures formed and the polarity of the solvent is evident. Preliminary spin-echo neutron scattering studies (SESANS) indicated the absence of any larger scale structures. Inelastic neutron spectroscopy (INS) studies demonstrated that the solvent is largely unaffected by gelation, but does reveal insights into the thermal history of the samples. Further neutron studies of this kind (particularly SESANS and INS) are warranted, and it is hoped that this work will stimulate others to pursue this line of research.

## Introduction

A detailed, molecular level understanding of the fundamental aspects of the spontaneous self-assembly and network formation of low molecular mass organogelators (LMOGS) is still elusive, although much research attempting to quantify the fundamental aspects of this fascinating phenomenon is underway [1–5]. A wide range of structurally diverse gelators have been identified, and in general, whilst a particular gelator functions for a small set of solvents, its gelling ability is not universal [3–11]. This lack of generality doubtless arises as there is generally no single unifying mechanism for gelation, which invariably involves a range of physical (non-covalent) interactions, such as hydrogen bonding, solvophobic effects and π–π interactions [11–34].

Our previous work has focused on the thermoreversible gelation of partially fluorinated liquids by a homologous series of chiral, non-racemic *bis*-(α,β-dihydroxy ester)s [35], in which the gelation character was found to depend on both solvent composition and the molecular structure of the gelator. The enthalpy of melting Δ*H*_m_ for a series of gelators was found to be positive, indicating an endothermic melting process, associated with the increase in entropy. Interestingly, the enthalpies were insensitive to both the solvent composition and the gelator chain length (G*_n_*, where *n* corresponds to the number of CH_2_-groups in the interheadgroup spacer less 2), suggesting that gelation is a feature dominated by the common end-group structural motif. A gelation mechanism based on hydrogen bonding of the end-group was confirmed by IR and circular dichroism (CD) spectroscopic characterisation. The specific stereochemistry of the gelator end-groups is a crucial factor, providing an obvious analogy to molecular recognition phenomena.

Small-angle neutron scattering provided a detailed insight into the gelator self-assembled structures, with data being best interpreted with a Kholodenko–Dirac worm model, comprising a flexible assembly of rodlike structures, in which the balance between flexibility and rigidity is defined by the parameter *m*. For the fluorinated systems, *m* = 4, indicating a rather rigid structure, with a Gaussian cross-section of 25–40 Å depending on gelator concentration and structure. The rodlike segments were typically hundreds of Å in length, suggesting a stacked geometry that was later confirmed with CD spectroscopy. With an increase in temperature, these structures simply “melt”, i.e., the size of the structure is largely invariant until the gel temperature is reached. Interestingly, whereas the size and shape of the scatterers was not found to vary significantly with gelator concentration, the scattering intensity did increase with gelator concentration indicating that the number of scatterers increases, leading to the stiffer gels implied by the concomitant increase in *T*_gel–sol_ observed.

These gelators form gels in a wide range of solvents, a rather serendipitous and unusual discovery. It is that observation that is elaborated here, and in particular, our focus is to probe the structures present in these gels. To this end, small-angle neutron scattering (SANS) has been used, supplemented in a small number of cases, with inelastic neutron spectroscopy (INS), spin-echo neutron scattering (SESANS), and pulsed-gradient spin-echo (PGSE-)NMR measurements.

## Results and Discussion

A series of *bis*-(α,β-dihydroxy ester)s have been found to gel a wide range of solvents at a solvent-specific concentration (C_gelator_), typically a few mg per ml, e.g., for G_6_ 3.8 mg ml^−1^ in toluene, 7.1 mg ml^−1^ in dichloromethane, 4.8 mg ml^−1^ cyclohexane, 4.1 mg ml^−1^ in chloroform/hexane (90% hexane) and 1.8 mg ml^−1^ in water-rich (75%) ethanol/water mixtures. The gels formed from water-rich systems and cyclohexane showed varying degrees of opacity, depending on the gelator concentration, but all other systems were transparent. Of the common solvents tested, the only non-gelling system was with acetonitrile. As is evident from this list, these liquids encompass highly polar liquids, nonpolar liquids, and those that are strongly hydrophobic, and at such low gelator concentrations (<10 mg ml^−1^). It is surprising, therefore, that a single gelator can gel such a wide range of liquids. With the exception of the water/ethanol system, there is a clear correlation of the order of the minimum gelator concentration required (water/ethanol < cyclohexane ≈ toluene < dichloromethane ≤ acetonitrile) with the dielectric constants (hexane ≈ cyclohexane ≈ toluene < dichloromethane < ethanol < acetonitrile ≤ water), suggesting that the gelation mechanism is driven by the polarity (and therefore, the strength of the hydrogen bonding) sensed by the gelator headgroups.

**Figure 1:**
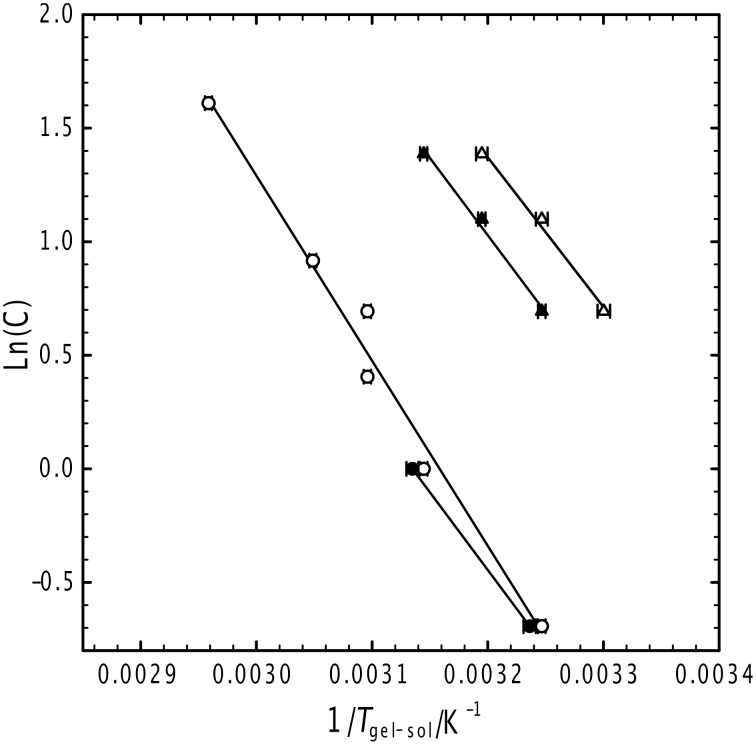
Schröder–van Laar analysis for the melting of toluene (open symbols) and ethanol/water (filled symbols) gels formed from G_6_ (circles) and G_8_ gelators (triangles).

**Scheme 1:**
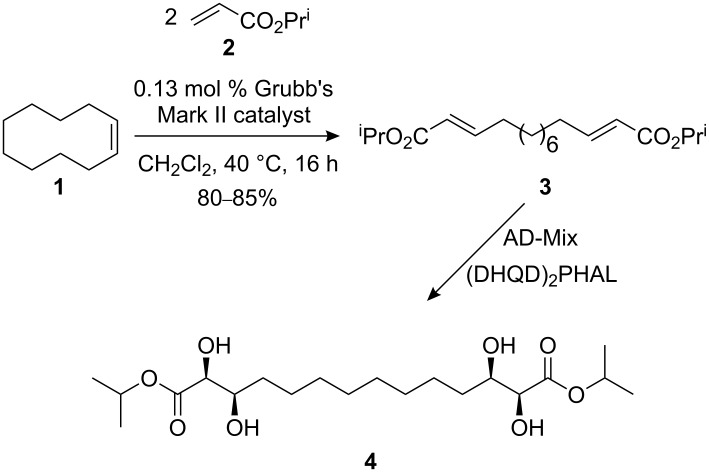
Synthesis of hydrogenous gelators.

Figure 1 presents a thermodynamic analysis of gelation for a selection of the gels examined here, focusing specifically on the toluene and ethanol/water gels for gelators G_6_ and G_8_ (see Scheme 1 in the Experimental section). In all cases, the gelation temperature increases with concentration, with the toluene gels melting at higher temperatures for the same concentration of gelator, a trend that is more pronounced for the G_6_ gelator. The higher melting temperature of the toluene gels, typically 20 °C, indicates that the gel is considerably more stable. The slopes of the data in this Schröder–van Laar representation correspond to the enthalpy of melting, and these were found to be 70 (±5) kJ mol^−1^ and 55 (±5) kJ mol^−1^ for the toluene and ethanol/water cases, respectively, and consistent with multiple hydrogen bonds between the headgroups, as found previously in the case of the fluorinated solvents [35]. For the cyclohexane systems, only a fragile gel is observed (even after several heat–cool cycles), over wide ranges of gelator concentration (5 < C_gelator_ < 50 mg ml^−1^) and temperature (25 < *T* < 55 °C), this representing the poorest performance of the gelator.

**Figure 2:**
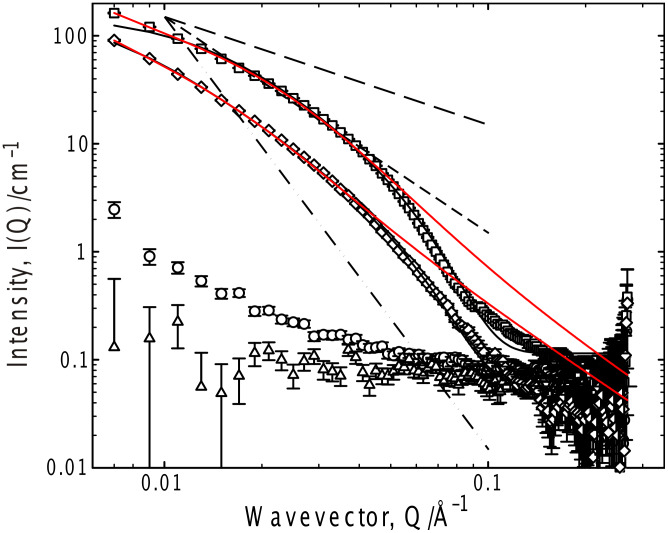
SANS from 50 mg ml^−1^ G_6_ in a range of solvents, 25 °C; *d*-acetone (circles), *d*-chloroform (triangles), 50% *d*-ethanol/D_2_O (squares) and *d*-toluene (diamonds). Fits to the Kholodenko–Dirac worm model are superimposed on the data as solid black lines, whereas the solid red lines are best attempts to describe the data by a two correlation length model. Limiting behaviors of Q^−1^, Q^−2^ and Q^−4^ are also shown.

**Figure 3:**
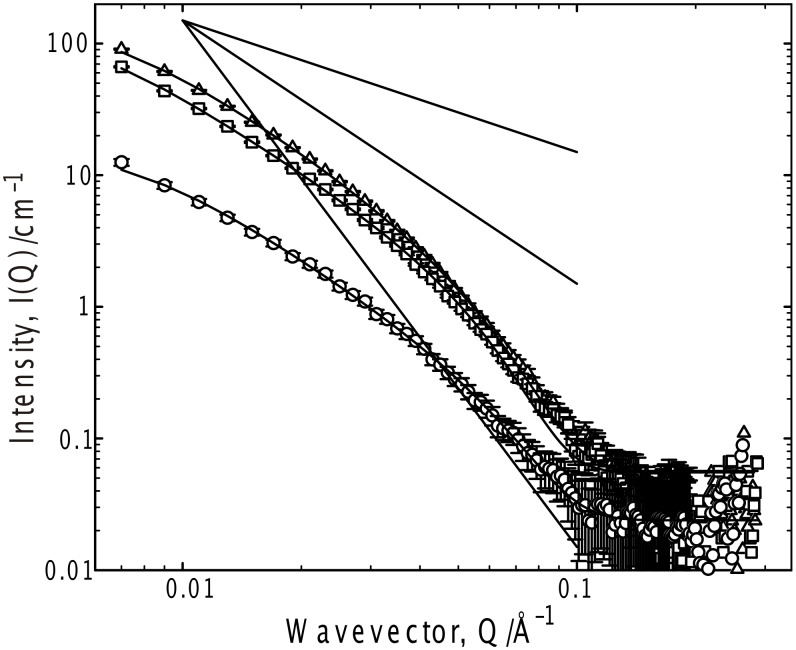
SANS from G_6_ 25 °C in *d*-toluene as a function of G_6_ concentration; 5 mg ml^−1^ (circles), 10 mg ml^−1^ (squares) and 50 mg ml^−1^ (triangles). Fits to the Kholodenko–Dirac worm model are superimposed on the data. Limiting behaviors of Q^−1^, Q^−2^ and Q^−4^ are also shown.

**Figure 4:**
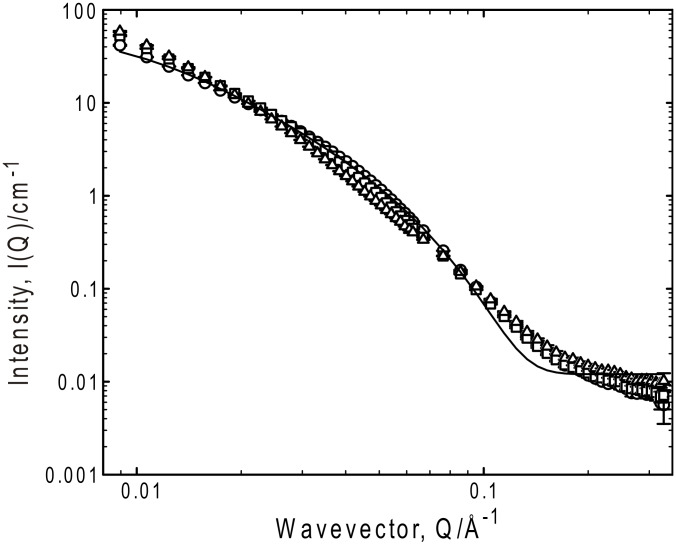
SANS from a series of homologous gelators, G_5_ (circles), G_6_ (squares) and G_8_ (triangles) in *d-*toluene, all at 50 mg ml^−1^ and 25 °C. Selected fits to the Kholodenko–Dirac worm model are superimposed on the data.

**Figure 5:**
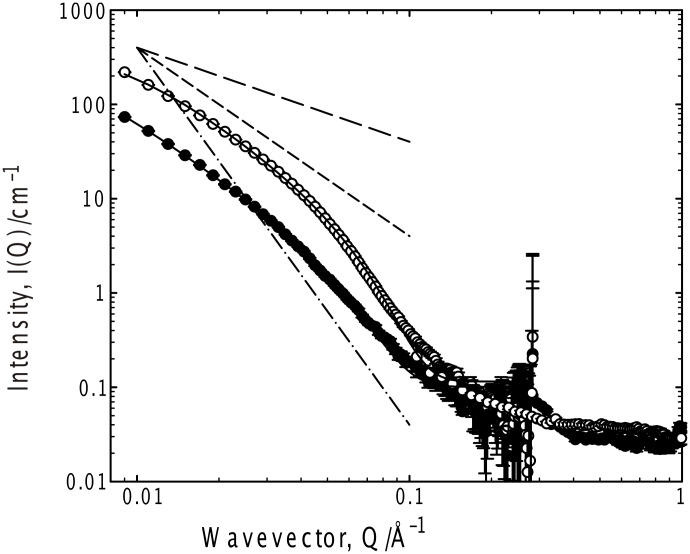
SANS from 50 mg ml^−1^ G_6_ in *d-*toluene, 25 °C; hydrogenous gelator (empty symbols) and partially deuterated gelator (filled symbols). Fits to the Kholodenko–Dirac worm model are superimposed on the data. Limiting behaviors of Q^−1^, Q^−2^ and Q^−4^ are also shown.

**Figure 6:**
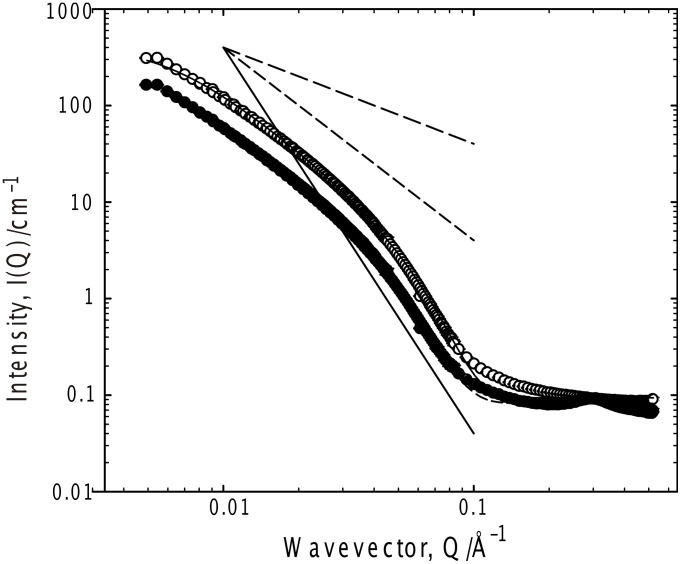
SANS from 35 mg ml^−1^ G_6_ in 75% *d-*ethanol/25% D_2_O, 37 °C; hydrogenous gelator (empty symbols) and partially deuterated gelator (filled symbols). Fits to the Kholodenko–Dirac worm model are superimposed on the data. Limiting behaviors of Q^−1^, Q^−2^ and Q^−4^ are also shown.

Representative SANS data are presented in Figures 2–6 from which the morphology of the structures can be extracted in terms of the nature of the solvent (Figure 2), the gelator concentration (Figure 3) and the gelator chain length (Figure 4). These studies have focused on toluene and cyclohexane gels, in which a “contrast variation” approach has been adopted to probe the internal structure of the gels (Figure 5 and Figure 6). The chloroform sample, at this gelator concentration, was not gelled (Figure 2) and no scattering is evident. Thus, we may conclude that there is no aggregation in these solutions that leads to structures large enough to scatter neutrons, i.e., the gelator molecularly dissolves.

The most common macroscopic structural arrangement formed by (chiral) gelators are fibrils formed from a stacking of the gelator molecules [15–16,19,22,27–28,34,36–40], which exhibit “signature” intensity vs. wave–vector (Q) relationships, viz Q^−1^ (rod) at low Q becoming Q^−4^ (Porod scattering from smooth surfaces, characteristic of a well-defined aggregate) at higher Q in conjunction with local maxima or oscillations usually at higher Q arising from Bragg reflections or sharp interfaces, or a switch from a Q^−1^ to a Q^−2^ dependencies on a double logarithmic I(Q) versus Q plot [13,41–49]. With the exception of the acetone sample, which shows very weak scattering, all the systems show a smooth transition from a simple Q^−1^ dependence indicative of rodlike structures, into the Q^−4^ expected for well-defined objects. This latter dependence (Q^−4^) is most evident in 75% D_2_O/*d-*ethanol, implying the structure in this case is more particle-like, consistent with the fragility (and appearance) of this gel. Clearly, existing frameworks for analyzing such data are not appropriate here.

In order to identify an appropriate theoretical framework with which to analyze these data, it is important to probe for any interaction between the structures – to ascertain whether the data may be analyzed merely in terms of their morphology or if there is an additional contribution from inter aggregate correlations. This is most easily assessed by recording the scattering from a series of gels as a function of the gelator concentration. The concentration dependence of the scattering from G_6_ was therefore studied over a wide range of concentration, and representative data is presented for the toluene system in Figure 3. The toluene system was chosen as there is appreciable scattering in this system, even just above the minimum gelation concentration.

From this system presentation, it is obvious that the functional form of the scattering does not follow a simple Q dependence at any concentration of gelator and that this complex functional form is invariant with concentration. This observation therefore, allows the data analysis to ignore incorporation of a term describing an interaction between the aggregates. Further, one may conclude that the changes in scattering arise from an increase in the number of structures, with perhaps subtle changes in their size or morphology (at least over this range), i.e., gelation is akin to the simple self-assembly process demonstrated by surfactants, and is a consequence of a cooperative process. Given the morphology of the scatterers, this is most likely to arise due to growth along the long axis (elongation) of the structures.

An analysis protocol has therefore been adopted that reflects the smooth transition in Q dependence observed in these systems, viz a flexible wormlike structure, parameterized by the Kholodenko–Dirac model. This model is based on a Gaussian coil comprising multiple (*m*) cylindrical elements of statistical length (*ℓ*) and radius (*R_Ax_*). The parameter *m* can be considered a measure of the balance of the Gaussian to rigid rod character – when *m* is large, and both *ℓ* and *R_Ax_* small, the limiting behavior of this model is effectively that of a Gaussian Debye coil, whereas when *m* is small and *ℓ* large, the limiting behavior is that of a rigid rod.

Representative fit parameters are given in Table 1, and the model predictions overlaid on the experimental scattering in all the figures. The fitting is most sensitive to the radial cross-section of the structure (*R_Ax_*), invariably with a radius of 25–35 Å. Converting this Guinier radius to an equivalent cylindrical radius (multiply by √2), and if one considers a disc of thickness 1 Å and the volume of a gelator molecule to be 600 Å^3^, that disc contains on average 6 ± 1 molecules, consistent with the proposed number of hydrogen bonds between the end-groups of the gelator molecules [35]. Further, the universality of the radius would seem to be a feature of the gelator structure rather than the solvent, i.e., self-association driven by a molecular recognition process, as opposed to a classical aggregation such as that observed in surfactants. There is a negligible variation in the scattering behavior with increasing gelator length, as might be expected were a bilayer structure present. In all cases, the fitting procedure was tested to ensure that the best fit observed was a global minimum, especially since there is some coupling of the parameters *m* and *ℓ*.

**Table 1:** Parameters describing the fit to the SANS data using the Kholodenko–Dirac worm model.

[Gelator]/wt %	Solvent	Number of links m	Length of link, l Å	Radial cross-section *R_Ax_*/Å

[G_6_] = 0.5	toluene	30 (±2)	90 (±5)	28 (±1)
[G_6_] = 1.0	toluene	30 (±3)	110 (±8)	30 (±1)
[G_6_] = 5.0	toluene	55 (±5)	100 (±10)	30 (±2)
[G_6_] = 5.0	acetone	Does not fit to Kholodenko–Dirac worm model, simple rod Q dependence		
[G_6_] = 5.0	50% ethanol/water	4 (±0.2)	200 (±20)	33 (±2)
[G_6_] = 10.0	chloroform	13 (±2)	100 (±10)	25 (±1)
[G_6_] = 10.0	acetone	4 (±1)	220 (±8)	30 (±2)
[G_6_] = 10.0	ethanol	55 (±10)	85 (±10)	20 (±3)
*d-*[G_6_] = 5.0	toluene	55 (±10)	100 (±10)	30 (±3)
*h-*[G_6_] = 5.0	toluene	60 (±5)	80 (±10)	25 (±3)
*d-*[G_6_] = 5.0	25% ethanol/water	480 (±3)	32 (±2)	27 (±2)
*h-*[G_6_] = 5.0	25% ethanol/water	460 (±3)	30 (±2)	26 (±2)

The internal morphology of the structures may be elaborated by considering a partially deuterated gelator, in which the end-groups no longer contribute to the scattering, an experimental approach known as “contrast variation”. For both the toluene and 25% ethanol/water systems, there is no significant change in the form of the scattering (merely the intensity) when the partially (headgroup) deuterated gelator is used, although the relative change in intensity, typically a factor of 3 ± 0.5, is somewhat smaller than would be expected given a homogeneous structure. This indicates that the headgroups and the alkyl sections of the gelator exhibit a very similar morphology, but there is some spatial separation within the structure between the headgroups and the alkyl chain. Further, the deuterated gelator data show a weak peak at Q ≈ 0.3 Å^−1^, corresponding to a dimension of approx. 20 Å. Given that the length of the alkyl spacer is approximately 10 Å, the simplest interpretation of this distance is the characteristic length associated with the correlation between the (deuterated) end-groups of two end-on gelator molecules. If this arrangement were heavily populated in the aggregate, one would also expect to see a feature on the SANS around a Q commensurate with the 10 Å distance, i.e., Q = 0.6 Å^−1^, but the data are not of sufficient quality to state whether the features at this Q are significant or not. It should be noted that a core-shell morphology was also tested, but the peak could only be reproduced by assuming unphysical parameters – a 10 Å in length alkyl spacer and a 20 Å headgroup region – whilst the fit at low Q was poor.

If one considers the trends in the Gaussian coil-rigid rod character (*m*) and the length (*ℓ*) of the rods comprising the building blocks of the gelled structure, i.e., for *m*; fluorinated solvent ≈ acetone ≈ ethanol/water < chloroform < toluene, whereas for *ℓ*; chloroform ≈ fluorinated solvent ≈ toluene << ethanol/water ≈ acetone, it is clear that more Gaussian coil- or particlelike structures are evident when the hydrogen bonding is weaker.

The fitted parameters, combined with the absolute intensities, may be used to further elaborate the structure of the gelator assemblies. Taking the parameters listed in Table 1 and the absolute intensity, the number of worms per unit volume, N, may be calculated, along with the number of molecules per worm, and ultimately the number of gelator molecules per worm per unit length. For all the data presented here, this characteristic number is surprisingly invariant across the systems, typically 8 ± 2 molecules, not inconsistent with the estimate of the same parameter derived from the radius of the rodlike element (6 ± 1).

**Figure 7:**
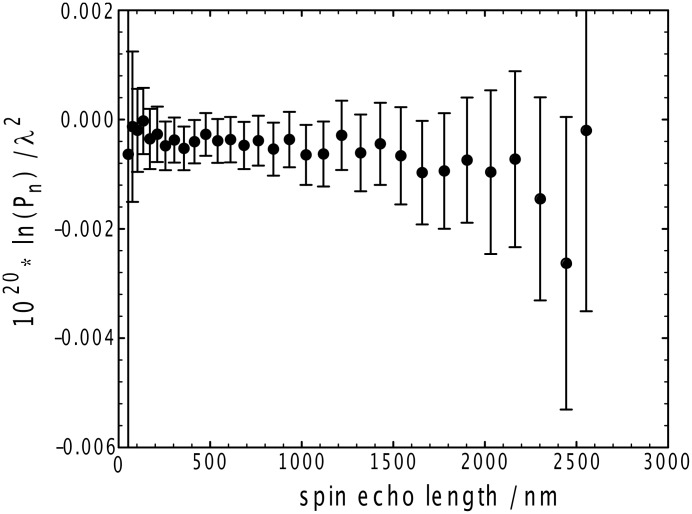
SESANS data for 50 mg ml^−1^ G_6_ in deuterated toluene, 25 °C.

A variation of the scattering experiment, SESANS, has been used to probe the existence of any structural order on a length scale greater than a few tens of nanometers up to several microns, Figure 7, for the toluene gels. In this experiment, polarized neutrons are used as a probe of long-range order. Polarized and nonpolarized neutrons probe structure differently, such that the ratio of their intensities is a measure of any structure present, over a distance scale defined by the evolution period used in the experiment. For the gels studied here, this ratio was constant at a value of unity, indicating a complete lack of higher order structure present in these systems, and one may conclude that there is no long-range ordering or association of the fibrils, i.e., they are randomly dispersed over these characteristic length scales.

**Figure 8:**
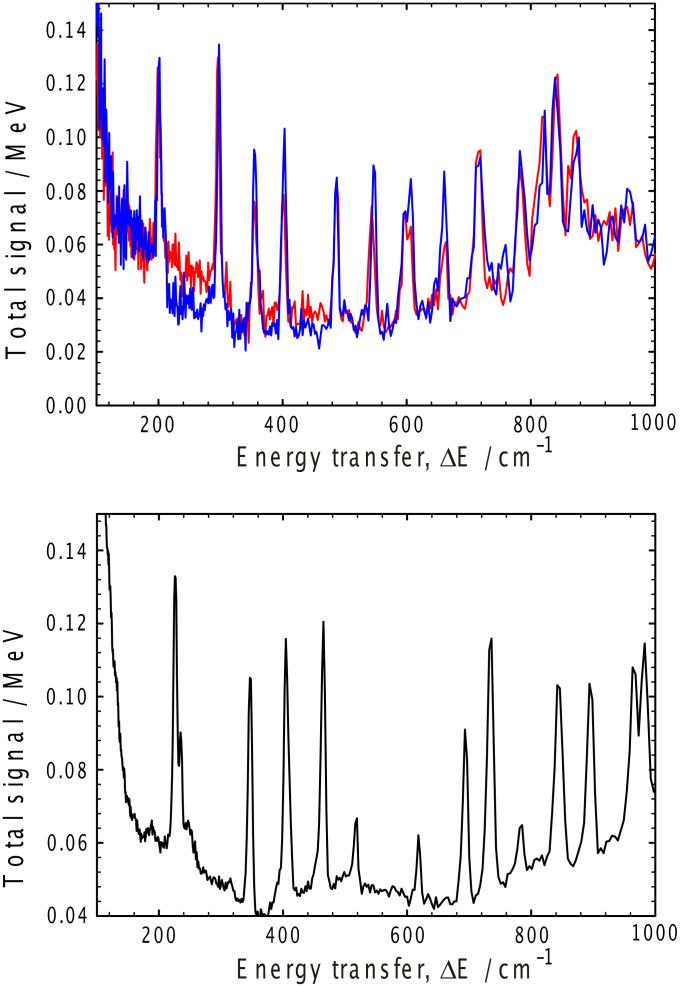
Inelastic neutron spectroscopy from toluene gels (upper Figure): red deuterated toluene/hydrogenous gelator, blue deuterated toluene/deuterated gelator, and the pure solvent (lower trace).

**Figure 9:**
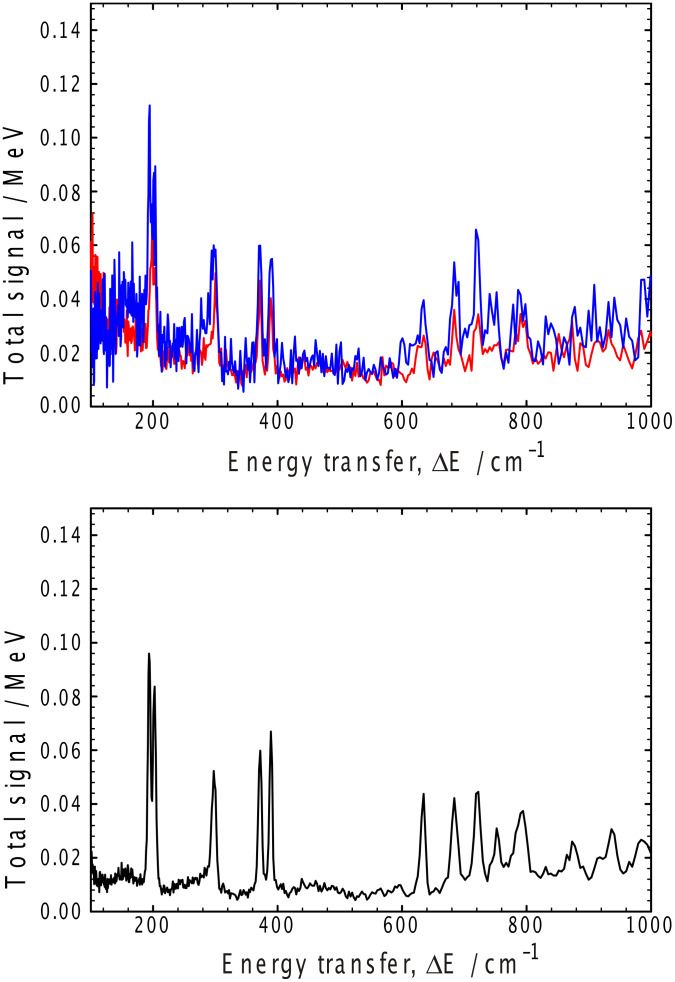
Inelastic neutron spectroscopy from cyclohexane gels (upper Figure): red deuterated cyclohexane/hydrogenous gelator, blue deuterated cyclohexane/deuterated gelator, and the pure solvent (lower Figure).

The characteristics of the solvent in these gels have been examined by inelastic neutron spectroscopy (INS), Figure 8 and Figure 9, for the toluene and cyclohexane gels, respectively. For the toluene gels, the observed gel spectra are dominated by the toluene spectrum (Figure 8). There are very subtle differences between the *h*- and *d*-gelator spectra, these being most obvious in the OH stretching region around 3000 cm^−1^ and in the low frequency region below 150 cm^−1^. However, the data in these regions are too broad and statistically too poor to extract any detailed interpretation, but it is comforting that the spectra are different in those regions of the spectrum where the peaks due to those functional groups that have been altered by deuteration would be expected.

The nine sharp features between 150 and 800 cm^−1^ arising from the *d-*toluene in the gelled samples are of considerably more interest, indicating that the average environment of the toluene methyl groups are significantly different. This difference may have a number of origins, one of which may be as a result of deuteration, but it is much more likely due to unavoidable thermal histories of the two samples (cooling rates, time elapsed since the initial freezing). Further, it is not possible to normalize the spectra such that all of these nine bands overlap completely with the same intensity in both spectra, again a consequence of differences in the samples rather than for example, spectrometer performance or gelation. Thus, it is probable that the differences in the spectra observed for the *d-*toluene gels arise as a result of two populations of coexisting solvent phases – the amorphous phase that has a structural arrangement of the molecules similar to that in the unstable β-toluene phase [50] and the more stable α-toluene phase – since the average barrier to methyl rotation in the amorphous phase is higher than the barrier heights in either the α- or β-phases [51]. Hence, when comparing two samples such as we do here, if the average barrier to methyl rotation is less in one sample than the second, the methyl group’s Debye–Waller factors that control that portion of the intensities in the observed bands arising from the methyl groups will differ, thereby accounting for the observed variations in the intensity of those bands involving most methyl group vibrations. A similar conclusion – that the solvent does not participate in the gelation mechanism – may be drawn from the cyclohexane data; again, the two spectra are very similar, the key differences being in the bands relating to the isopropyl dynamics.

Finally, the self-diffusion coefficients of the solvents (toluene, cyclohexane, water/ethanol) in these gels (at 25 °C) have been measured by PGSE-NMR (data not presented), and compared with the self-diffusion coefficients in the appropriate bulk solutions. A very slight retardation in the diffusion of the solvent is observed, consistent with the obstruction effect introduced by the assemblies of the gelator molecules, as the solvent molecules must diffuse around the structures, thereby increasing their diffusion path length.

## Conclusion

A diverse range of liquids has been successfully gelled with low concentrations of a low molecular weight gelator incorporating *bis*-(α,β-dihydroxy ester) end-group motifs. Gelation is caused by association of gelator molecules into stacks, due to intermolecular hydrogen bonding between the end-groups. The critical dimensions of these structures have been determined by analysis of neutron scattering data (SANS and SESANS), and are dependent on the strength of the intermolecular hydrogen bonding interaction. The strongest gels are formed in solvents where stronger intermolecular hydrogen bonds lead to longer segments and less flexible structures, whereas shorter, more flexible segments lead to a particlelike gelator structure which can only form weak gels. PGSE-NMR confirms retardation of solvent diffusion due to an obstruction effect. Preliminary INS data exhibit subtle differences providing an insight into the thermal history of the sample, but not the gelation mechanism.

## Experimental

### Materials

All solvents were of spectroscopic grade and used as received.

#### Synthesis of gelators

The omnigelator **4** was prepared as previously described [52] by sequential Grubbs double cross metathesis [53] between (*Z*)-cyclodecene (**1**) and two equivalents of isopropyl acrylate **2**, which routinely delivered 80–85% yields of the bisenoate **3**, exclusively as the (*E,E*)-isomer shown (Scheme 1), followed by an AD-mix double bishydroxylation with (DHQD)_2_PHAL as the chiral ligand [54].

**Scheme 2:**
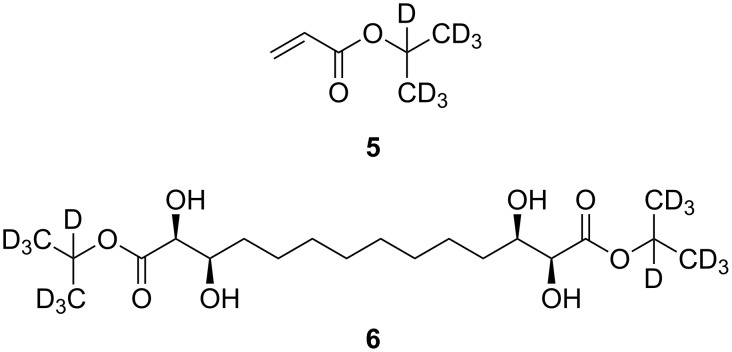
Structure of partially deuterated gelators.

The deuterated analogue **6**, Scheme 2, was prepared by using *d*_7_-isopropyl acrylate (**5**) in the initial cross metathesis step. The deuterated ester **5** was prepared from acryloyl chloride and *d*_7_-isopropanol (Et_3_N, CH_2_Cl_2_, 0→20 °C, 2 h); yields were essentially identical to the nondeuterated example, given that the deuterated acrylate **5** was carefully purified and was completely free of triethylamine, which is a very effective ligand and quench for the Grubbs Mark II metathesis catalyst. This was achieved by extensive washing during work-up (water, sat. aq NH_4_Cl, 1 M HCl, sat. aq K_2_CO_3_, water and brine) followed by distillation, bp ~40 °C at 17 mm Hg.

#### Gel formation

On a 0.5 g scale, the gelator and solvent were weighed directly into a screw top vial. A simple heat–cool cycle was necessary for several of these gels to ensure homogeneity, although several heterogeneous gels did form spontaneously at room temperature.

#### Determination of gelation temperature *T*_gel–sol_

Glass vials containing the samples were equilibrated in a temperature-controlled water bath and the temperature increased from 15 °C initially in 2 °C steps with a 30 min equilibration time at each temperature. On approaching the gelation temperature, smaller increments (0.5 °C) were adopted. The simplest measure of gelation – that the gel be stable to inversion [3] – was used to quantify the gel–sol behavior.

#### Small-Angle Neutron Scattering (SANS)

Small-angle neutron scattering (SANS) measurements were performed on either (a) the fixed-geometry, time-of-flight LOQ diffractometer (ISIS Spallation Neutron Source, Oxfordshire, UK) or (b) on the D11 diffractometer at the ILL, Grenoble. On LOQ, a white beam of radiation with neutron wavelengths spanning 2.2 to 10 Å were used to access a Q [Q = 4πsin(θ/2)/λ] range of 0.008 to 0.25 Å^−1^ (25 Hz), with a fixed sample-detector distance of 4.1 m. On D11, a neutron wavelength of 6 Å was employed to access a Q-range of approximately 0.005 to 0.50 Å^−1^, requiring three separate instrument configurations (sample-detector distances and collimation).

On both instruments, the samples were contained in 2 mm path length, UV-spectrophotometer grade, quartz cuvettes (Hellma) and mounted in aluminium holders on top of an enclosed, computer-controlled, sample chamber. Sample volumes were approximately 0.4 cm^3^. Temperature control was achieved through the use of a thermostatted circulating bath pumping fluid through the base of the sample chamber. Under these conditions a temperature stability of better than ±0.5 °C can be achieved. Experimental measuring times were approximately 40 min.

All scattering data were (a) normalized for the sample transmission, (b) background corrected using an empty quartz cell or one filled with the appropriate solvent (this also removes the inherent instrumental background arising from vacuum windows, etc) and (c) corrected for the linearity and efficiency of the detector response using the instrument-specific software package. The data were put onto an absolute scale by reference to the scattering from a partially deuterated polystyrene blend (LOQ) or 1 mm H_2_O.

The Kholodenko–Dirac wormlike chain model [9] has been used to analyse the SANS data. This approach is derived from a Gaussian coil model, where long thin rods are made of a succession of *m* cylindrical elements of statistical length *ℓ* and radius *R_Ax_*. The contour length of the chain, L, is equal to the product *m*·*ℓ*. The scattering intensity generated from Kholodenko–Dirac wormlike chains is proportional to two terms:

[1]
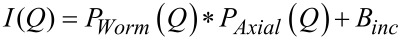


where *B_inc_* is the incoherent background. The Kholodenko–Dirac model therefore smoothly interpolates between the Gaussian coil and rigid rod predictions and the number of segments (*m*) forming the chain, and hence gives an indication regarding the flexibility of the chain. Smaller values of *m* correspond to stiffer chains. When *m* tends towards infinity, the scatterer adopts a flexible Gaussian random coil whereas when tending towards unity, a rigid rod is obtained.

For long thin rods

[2]
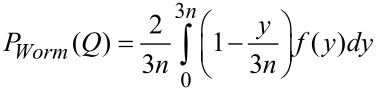


where for


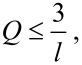



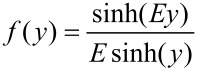


with


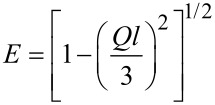


whereas for


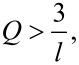



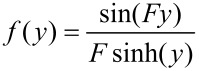


with


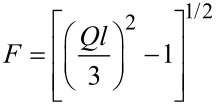


given that *m* is the number of chain elements, *l* the statistical chain element length (giving the total chain length L = *m·l*).

*P_Axial_* (Q) was adopted with a radial Guinier form, such as:

[3]
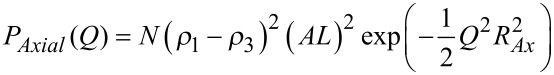


with *ρ*_1_ and *ρ*_3_ the scattering length densities for the worm and solvent, *N* worms per unit volume, *A* the cross sectional area and *R_Ax_* the cross sectional radius of the chain, assuming a Gaussian scattering density.

#### Inelastic Neutron Scattering (INS)

The samples, about 0.5 g each, were held in air-tight sample holders and rapidly cooled to 20 K in the TOSCA neutron spectrometer, ISIS Facility, the Rutherford Appleton Laboratory, Harwell Science and Innovation Campus, OX11 0QX, UK. TOSCA is a pulsed neutron, indirect geometry, low band-pass spectrometer with good spectral resolution (Δ*E*_t_/*E*_t_ ≈ 1–2%), further details are given elsewhere [55]. Data were collected for about 8 h and transformed into the conventional scattering law, S(*Q*,ω) (arbitrary units), vs. energy transfer, *E*_t_ (cm^-1^), using standard programs.
